# Clinical characteristics and etiology-specific outcome in pediatric hypertrophic cardiomyopathy

**DOI:** 10.1007/s00392-025-02703-7

**Published:** 2025-10-13

**Authors:** Felix Minette, Maximilian Klass, Nikita Meyer, Angela Merzweiler, Sebastian Burkart, Maja Hempel, Norbert Frey, Mirko Völkers, Matthias Gorenflo, Alexander Kovacevic, Christoph Sandmann

**Affiliations:** 1 Department of Pediatric Cardiology, Heidelberg University Hospital, Heidelberg, Germany; 2 Institute of Medical Informatics, Heidelberg University, Heidelberg, Germany; 3 Institute of Human Genetics, Heidelberg University, Heidelberg, Germany; 4 Department of Internal Medicine III (Cardiology, Angiology, and Pneumology), Center for Internal Medicine, Heidelberg University Hospital, Im Neuenheimer Feld 410, 69120 Heidelberg, Germany; 5 DZHK (German Center for Cardiovascular Research), Partner Site Heidelberg/Mannheim, Heidelberg, Germany; 6 Department of Genetics, Harvard Medical School, Boston, MA USA

**Keywords:** Hypertrophic cardiomyopathy, Cardiomegaly, Pediatrics, Genetic diseases, Inborn, Rare diseases

## Abstract

**Introduction:**

Childhood-onset cardiomyopathies are rare disease (incidence 1/100,000) presenting with diverse, potentially severe phenotypes. The etiologies range from idiopathic/sarcomeric forms to syndromic diseases, neuromuscular disorders, and inborn errors of metabolism, but cause-specific outcomes remain incompletely understood. This study analyzed the clinical course of a large cohort of children with hypertrophic cardiomyopathy (HCM), stratified by etiology.

**Methods:**

Patients clinically diagnosed with HCM before 18 years of age at Heidelberg University Hospital, Germany (2000–2024) were included (*n* = 146). The clinical data were compiled by the Medical Data Integration Center and supplemented by manual data extraction. Outcomes included survival, myectomy, ICD and PPM implantation, arrhythmias, heart transplantation, cardiac arrest, and echocardiographic features at first presentation.

**Results:**

Of 146 patients, 31.5% (*n* = 46) were followed into adulthood. HCM etiologies included idiopathic/sarcomeric (37%, *n* = 54), inborn errors of metabolism (21.2%, *n* = 31), RASopathy (15.7%, *n* = 23), neuromuscular disorders (6.8%, *n* = 10), other syndromic (6.2%, *n* = 9), and other (13%, n = 19). Diagnosis was made in infancy (< 1 year) (47.3%, *n* = 69), childhood (1–18 years) (40.4%, *n* = 59), or was confirmed before age 18 without specific timing available (12.3%, *n* = 18). Early diagnosis correlated with syndromic and multisystem disease. The echocardiographic findings and clinical outcomes varied by etiology. During a mean follow-up of 13.6 ± 10.5 years, 11% (*n* = 16) died, with 62.5% (*n* = 10) of deaths occurring within the first two years of life. Survival was highest in idiopathic/sarcomeric HCM (96.7%) and lower in neuromuscular (85.7%), syndromic (76.2%), inborn errors of metabolism (70.5%), RASopathy (57.8%), and other forms (54.2%). Death frequently involved non-cardiovascular causes. Infants had higher early mortality, which normalized among those surviving beyond two years. In idiopathic/sarcomeric HCM, outcomes did not differ between those diagnosed in infancy versus later childhood. Reduced ejection fraction and elevated NT-proBNP levels were predictive of mortality, while the use of Class IV anti-arrhythmics was associated with improved survival.

**Conclusions:**

The results of this analysis show significant variability of outcomes by HCM subtype in children. Idiopathic/sarcomeric and neuromuscular disease-associated HCM had the best prognosis, while other non-idiopathic/non-sarcomeric forms of HCM showed worse outcomes. Pediatric HCM presents with diverse underlying causes, unique phenotypes, and clinical trajectories, requiring tailored treatment approaches.

**Graphical Abstract:**

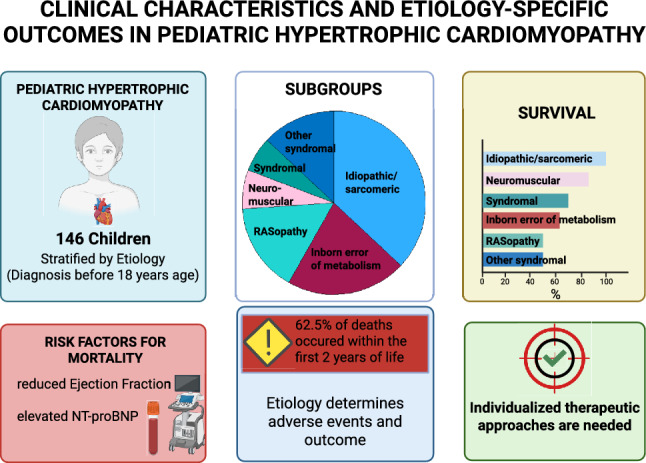

**Supplementary Information:**

The online version contains supplementary material available at 10.1007/s00392-025-02703-7.

## Introduction

Childhood-onset hypertrophic cardiomyopathy (HCM) is a rare disease with an estimated annual incidence of < 1/100,000 that is associated with substantial mortality [1–8]. Pediatric HCM can be caused by diverse underlying etiologies distinct from primary HCM (caused by pathogenic variants in genes that encode the sarcomere apparatus or are non-syndromic idiopathic) [3, 5], including RASopathies [5, 9, 10], inborn errors of metabolism [5, 11], neuromuscular disorders [5, 12], other syndromic diseases [5], and a diverse group of other, less common causes [5, 13].

The clinical manifestations of pediatric HCM are highly variable. Idiopathic/sarcomeric HCM is typically characterized by isolated septal hypertrophy and carries a high risk of life-threatening ventricular arrhythmias [3, 6, 14]. In contrast, the non-sarcomeric forms often involve a broader spectrum of cardiac abnormalities and are frequently associated with multisystem involvement, which can significantly impact prognosis [10–12]. Despite these distinctions, cause-specific outcomes in pediatric HCM remain incompletely defined [5]. A detailed understanding of the clinical features and disease course across different etiologic subgroups is essential, as each may exhibit distinct phenotypes, trajectories, and underlying pathophysiological mechanisms, requiring a tailored approach to diagnosis and treatment.

This study analyzed the clinical course of a large cohort of children with HCM from a tertiary care pediatric cardiology center, stratified by underlying etiology. By analyzing the clinical characteristics, disease trajectories, and outcomes across subgroups, it highlights differences in clinical course and prognosis between subgroups and emphasizes the need for personalized management strategies in pediatric HCM.

## Results

### Demographics

**Fig. 1:**
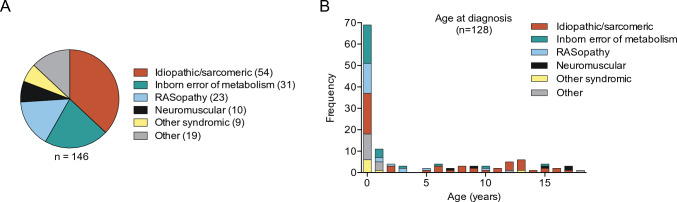
Pediatric HCM cohort classification by underlying cause. **A**, Pie chart indicating the relative frequencies of specific causes for HCM according the defined categories in this cohort. **B**, Bar graph showing the age at diagnosis in years, stratified by underlying etiology

One-hundred-and-forty-six patients diagnosed with HCM during childhood (< 18 years of age) were included, of whom 63 (43.2%) were female (significantly different sex distribution, *p* < 0.01, *t*–test). The patients were divided into subgroups by underlying cause: 1) idiopathic/sarcomeric (*n* = 54, 37.0%), 2) inborn error of metabolism (*n* = 31, 21.2%), 3) RASopathy (*n* = 23, 15.8%), 4) neuromuscular (*n* = 10, 6.8%), 5) other syndromic disease (*n* = 9,) 6.2%, and 6) other (*n* = 19, 13.0%) (Fig. 1A). The patients were classified as “idiopathic” if no signs of other underlying HCM-causing disease were present in the patient’s clinical history or genetic testing. The mean age of diagnosis of HCM was 3.8 (SD 5.59) years. 69 (53.9%) patients were diagnosed under the age of 1 year (Fig. 1B). Patient demographics are summarized in Table 1. A full list of each included patient and its classification according to the underlying etiology can be found in Supplementary Table 1.

**Table 1:** Demographics at baseline

Categories			Group							
			Total (*n* = 146)	Idiopathic/ sarcomeric (*n* = 54)	Inborn error of metabolism (*n* = 31)	RASopathy (*n* = 23)	Neuromuscular disorders (*n* = 10)	Other syndromic (*n* = 9)	Other (*n* = 19)	*p* value (Fisher Freeman Halton Exact Test)
Phenotype	HCM	n	143	54	31	23	10	8	17	**0.018**
		Proportion (%)	97.9%	00.0%	100.0%	100.0%	100.0%	88.9%	89.5%	
	HCM + LVNC	n	3	0	0	0	0	1	2	
		Proportion (%)	2.1%	0.0%	0.0%	0.0%	0.0%	11.1%	10.5%	
Gender	Female	n	63	14	16	13	6	4	10	**0.043**
		Proportion (%)	43.2%	25.9%	51.6%	56.5%	60.0%	44.4%	52.6%	
	Male	n	83	40	15	10	4	5	9	
		Proportion (%)	56.8%	74.1%	48.4%	43.5%	40.0%	55.6%	47.4%	
Age	< 1 year	n	69	19	18	14	0	6	12	**0.009**
		Proportion (%)	53.9%	39.6%	64.3%	66.7%	0.0%	75.0%	66.7%	
	> 1 year	n	59	29	10	7	5	2	6	
		Proportion (%)	46.1%	60.4%	35.7%	33.3%	100.0%	25.0%	33.3%	
	Childhood of unknown age	n	18	6	3	2	5	1	1	
		Proportion (%)	12.8%	4.1%	2.1%	1.4%	3.4%	0.7%	0.7%	
Family history	Unknown	n	47	14	9	9	4	3	8	**0.085**
		Proportion (%)	32.2%	25.9%	29.0%	39.1%	40.0%	33.3%	42.1%	
	Yes	n	17	14	2	0	0	0	1	
		Proportion (%)	11.6%	25.9%	6.5%	0.0%	0.0%	0.0%	5.3%	
	No	n	82	26	20	14	6	6	10	
		Proportion (%)	56.2%	48.1%	64.5%	60.9%	60.0%	66.7%	52.6%	
Consanguineous	No/missing	n	137	53	24	23	10	9	18	**0.008**
		Proportion (%)	93.8%	98.1%	77.4%	100.0%	100.0%	100.0%	94.7%	
	Yes	n	9	1	7	0	0	0	1	
		Proportion (%)	6.2%	1.9%	22.6%	0.0%	0.0%	0.0%	5.3%	

Significant differences (*p* < 0.05) are highlighted in bold

### Genetics

Genetic testing was performed in 66 patients (45.2%). Of these, results were available for 35 (42.4%) cases, with a pathogenic or likely pathogenic variant identified in 22 (62.9%) children, and a variable of unknown significance or conflicting classification in 6 children (17.1%). In this cohort, genetic testing was not routinely performed in the idiopathic/sarcomeric group, as formal recommendations for pediatric HCM genetic testing were only introduced during the latter part of the study period (recommendation for genetic testing as of 2023/2024^15–17^). Consequently, testing rates differed significantly across subgroups (*p* < 0.001, Pearson chi-square), with lower testing frequency in idiopathic/sarcomeric patients and higher rates among those with inborn errors of metabolism, RASopathies, and neuromuscular disorders (Supplementary Table 2). The genetic testing results are shown in Supplementary Table 3.

### Echocardiographic characteristics

Echocardiographic data were analyzed from the first echocardiographic examination at assessment in the study center (Table 2). While echocardiographic parameters were overall heterogeneous across groups, the idiopathic/sarcomeric subgroup presented with higher overall z-scores for septal thickness whereas other subgroups, particularly children with an underlying inborn error of metabolism, showed more pronounced hypertrophy of the left ventricular free wall. In addition, cardiac hypercontractility was observed in children with idiopathic/sarcomeric, RASopathy-associated, or other syndromal forms of HCM, whereas this feature was less apparent in those with inborn errors of metabolism or neuromuscular disease. There was a high variance for LVOT pressure gradient among patients, with overall higher values in idiopathic/sarcomeric, RASopathies, inborn error of metabolism and other syndromic disease compared to neuromuscular disorders and other rare causes of HCM. In addition, there was significant difference for MV A Vmac among subgroups. Most other echocardiographic parameters showed trends toward differences among subgroups, suggesting etiology-specific features and limited statistical power in this cohort to detect significant variation.

**Table 2:** Echocardiographic findings at first presentation to the study center

Group	idiopathic/sarcomeric (*n* = 54)		inborn error of metabolism (*n* = 31)		RASopathy (*n* = 23)		neuromuscular disorders (*n* = 10)		other syndromic (*n* = 9)		other (*n* = 19)		*p*-value (Kruskal–Wallis test)
Echocardiography parameter	Valid (n)	Mean (Std. Deviation)	Valid (n)	Mean (Std. Deviation)	Valid (n)	Mean (Std. Deviation)	Valid (n)	Mean (Std. Deviation)	Valid (n)	Mean (Std. Deviation)	Valid (n)	Mean (Std. Deviation)	
IVSd (z-score Detroit)	48/54	7.83 (6.73)	30/31	4.33 (3.17)	19/23	5.17 (2.92)	6/10	3.86 (2.98)	9/9	5.40 (4.95)	15/19	4.06 (1.88)	0.059
LVIDd (z-score Detroit)	49/54	− 1.13 (2.73)	30/31	− 0.16 (2.13)	19/23	− 2.46 (1.14)	7/10	− 1.88 (1.24)	9/9	− 2.74 (1.77)	15/19	− 1.99 (2.26)	** < 0.001**
LVPWd (z-score Detroit)	48/54	2.04 (3.57)	30/31	3.74 (3.50)	18/23	1.90 (2.18)	7/10	2.55 (1.85)	9/9	1.81 (2.03)	15/19	1.96 (2.33)	**0.022**
IVSs (z-score Detroit)	48/54	5.06 (4.07)	30/31	2.43 (2.18)	19/23	3.59 (2.01)	7/10	2.62 (2.18)	8/9	3.18 (2.44)	15/19	2.72 (1.78)	**0.020**
LVIDs (z-score Detroit)	48/54	− 1.74 (2.60)	30/31	0.23 (2.82)	19/23	− 3.76 (1.79)	7/10	− 1.38 (2.12)	9/9	− 4.92 (4.92)	15/19	− 2.46 (2.35)	0.052
LVPWs (z-score Detroit)	47/54	1.61 (2.71)	30/31	2.96 (2.23)	18/23	2.30 (1.55)	7/10	1.95 (2.77)	8/9	2.13 (2.16)	15/19	2.23 (1.73)	0.103
FS (M-mode)	51/54	43.47 (8.80)	30/31	36.36 (12.74)	19/23	51.73 (11.33)	8/10	37.86 (12.81)	9/9	54.54 (17.65)	16/19	45.81 (13.93)	** < 0.001**
EF (M-mode, Teich)	46/54	73.01 (10.98)	27/31	64.68 (16.80)	17/23	78.86 (18.36)	6/10	64.85 (20.70)	6/9	83.73 (17.56)	13/19	70.27 (24.34)	**0.004**
LVOT pressure gradient (mmHg)	15/54	17.43 (13.80)	3/31	13.88 (19.60)	7/23	22.61 (14.36)	2/10	3.49 (2.38)	3/9	26.8 (15.76)	3/19	5.39 (3.75)	**0.097**
LVOT Vmax (m/s)	13/54	1.81 (0.78)	3/31	1.44 (1.35)	5/23	1.97 (0.84)	2/10	0.91 (0.32)	3/9	2.50 (0.74)	2/19	0.90 (0.28)	0.119
MV E Vmax (cm/s)	25/54	110.37 (38.89)	12/31	101.02 (27.61)	9/23	119.20 (30.18)	3/10	78.67 (20.55)	4/9	110.30 (55.83)	9/19	100.70 (33.40)	0.385
MV A Vmax (cm/s)	24/54	65.77 (34.88)	11/31	101.02 (27.61)	9/23	109.29 (41.18)	3/10	42.77 (6.31)	4/9	63.15 (35.21)	9/19	76.26 (34.15)	**0.038**
MV E/A	24/54	1.96 (0.82)	11/31	80.30 (35.67)	9/23	1.25 (0.60)	3/10	1.80 (0.20)	4/9	3.55 (4.91)	9/19	1.50 (0.66)	0.128
MV V2 max (cm/s)	2/54	232.65 (29.91)	3/31	1.50 (0.77)	0/23	N/A	0/10	N/A	0/9	N/A	0/19	N/A	0.083
MV max PG (mmHg)	2/54	21.85 (5.59)	3/31	128.93 (24.02)	0/23	N/A	0/10	N/A	0/9	N/A	0/19	N/A	0.083
MV V2m (cm/s)	2/54	132.60 (68.02)	3/31	6.80 (2.61)	0/23	N/A	0/10	N/A	0/9	N/A	0/19	N/A	0.248
MV MPG (mmHg)	3/54	6.80 (6.08)	3/31	69.10 (27.01)	1/23	2.20 (N/A)	0/10	N/A	0/9	N/A	0/19	N/A	0.361
MV V2 VTI (cm)	2/54	86.40 (5.09)	3/31	2.30 (1.39)	0/23	N/A	0/10	N/A	0/9	N/A	0/19	N/A	0.083
PV Vmax (cm/s)	7/54	113.04 (25.71)	4/31	20.80 (6.48)	3/23	244.60 (212.07)	1/10	80.90 (N/A)	2/9	197.95 (48.30)	3/19	134.50 (2.74)	0.093
PV max PG (mmHg)	7/54	5.31 (2.55)	4/31	109.75 (21.42)	3/23	36.07 (51.77)	1/10	2.60 (N/A)	2/9	16.15 (7.71)	3/19	7.23 (0.31)	0.083
LA D (z-score Detroit)	6/54	1.65 (1.70)	3/31	4.98 (1.92)	2/23	3.40 (0.00)	0/10	N/A	1/9	2.40 (N/A)	3/19	0.16 (1.28)	0.167
LA / Ao	22/54	1.44 (0.24)	14/31	1.04 (0.27)	9/23	1.43 (0.18)	1/10	1.30 (N/A)	2/9	1.65 (0.21)	10/19	1.39 (0.38)	N/A
Ao / LA	4/54	0.68 (0.16)	1/31	1.28 (0.16)	1/23	0.57 (N/A)	0/10	N/A	1/9	0.69 (N/A)	2/19	0.56 (0.15)	N/A

Significant differences (*p* < 0.05) are highlighted in bold

### Outcomes

The mean follow-up duration was 13.67 (SD 10.53) years. Clinical outcomes assessed included myectomy, implantable cardioverter-defibrillator (ICD), permanent pacemaker (PPM) implantation, cardiac arrest, ventricular assist device (VAD) implantation, heart transplantation and death (Table 3). Myectomy was performed in 31 (21.2%) patients at a mean age of 10.76 (SD 8.17) years. ICD implantation was done in 18 patients (12.3%) at a mean age of 14.45 (SD 10.66) years and 3 patients (2.1%) received a PPM (mean age 10.17 years, SD 9.63). The cardiac arrest was recorded in 23 (15.8%) patients, with mean age of 10.57 (SD 9.11) years. A heart transplantation was performed in 6 (4.1%) patients and no patient received a VAD. Age at cardiac transplantation was available for two patients, with a mean age of 15.8 years. Death occurred in 16 (11%) patients. The mean age at death was 7.37 (SD 10.41) years with 62.5% (*n* = 10) of deaths occurring within the first two years of life. While the incidence of cardiac arrest (*p* = 0.743), myectomy (*p* = 0.705), and PPM implantation (*p* = 0.837) did not differ between subgroups, there were significant differences in the incidence of ICD implantation (*p* = 0.031), heart transplantation (*p* =  < 0.001) and death (*p* = 0.030) (Table 3).

**Table 3:** Cause-specific outcome of childhood-onset hypertrophic cardiomyopathy

Categories				Group														
				Total (*n* = 146)		Idiopathic/sarcomeric (*n* = 54)		Inborn error of metabolism (n = 31)	RASopathie (*n* = 23)		Neuromuscular disorders (*n* = 10)		Other syndromic (*n* = 9)		Oher (*n* = 19)		*p* value (Fisher Freeman Halton Exact Test)	
Death	Yes	n	16		1		4			4		1		2		4		**0.030**
		Proportion (%)	11.0%		1.9%		12.9%			17.4%		10.0%		22.2%		21.1%		
	No	n	130		53		27			19		9		7		15		
		Proportion (%)	89.0%		98.1%		87.1%			82.6%		90.0%		77.8%		78.9%		
Cardiac arrest	None recorded	n	123		45		25			19		10		7		17		0.743
		Proportion (%)	84.2%		83.3%		80.6%			82.6%		100.0%		77.8%		89.5%		
	Cardiac arrest	n	23		9		6			4		0		2		2		
		Proportion (%)	15.8%		16.7%		19.4%			17.4%		0.0%		22.2%		10.5%		
Heart transplant	Yes	n	6		4		0			1		0		0		1		0.705
		Proportion (%)	4.1%		7.4%		0.0%			4.3%		0.0%		0.0%		5.3%		
	No	n	140		50		31			22		10		9		18		
		Proportion (%)	95.9%		92.6%		100.0%			95.7%		100.0%		100.0%		94.7%		
Myectomy	Yes	n	31		21		1			7		0		2		0		** < 0.001**
		Proportion (%)	21.2%		38.9%		3.2%			30.4%		0.0%		22.2%		0.0%		
	No/unknown	n	115		33		30			16		10		7		19		
		Proportion (%)	78.8%		61.1%		96.8%			69.6%		100.0%		77.8%		100.0%		
ICD	Yes	n	18		12		1			4		0		1		0		**0.031**
		Proportion (%)	12.3%		22.2%		3.2%			17.4%		0.0%		11.1%		0.0%		
	No	n	128		42		30			19		10		8		19		
		Proportion (%)	87.8%		77.8%		96.8%			82.6%		100.0%		88.9%		100.0%		

Significant differences (*p* < 0.05) are highlighted in bold

**Fig. 2:**
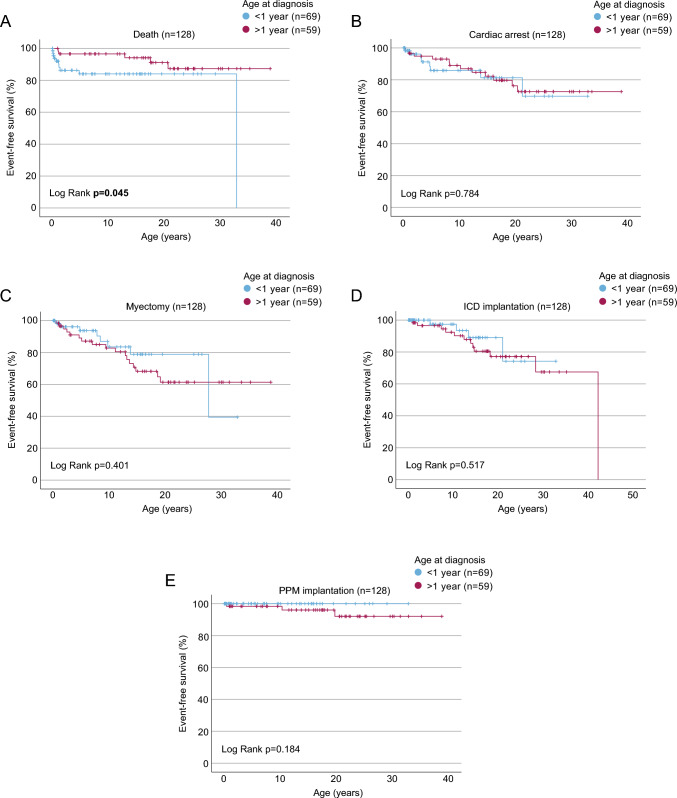
Outcome of pediatric HCM diagnosed before or after 1 year of age. Hazard free survival by Kaplan Meier analysis for the endpoints **A** death, **B** cardiac arrest, **C** myectomy, **D** ICD implantation, or **E** PPM implantation, stratified by age at diagnosis < 1 or > 1 year of age. Exact information of date at diagnosis was available for 128 children, *n* = 69 < 1 year and *n* = 59 > 1 year. Patients without exact information on age at diagnosis were excluded from the analysis. Statistical analysis represents the p-value of a log-rank test. *p* values reaching the pre-defined statistical significance threshold of 0.05 are printed in bold

Hazard free survival was analyzed by Kaplan–Meier analysis for the endpoints death, cardiac arrest, myectomy, ICD or PPM implantation. Analysis for heart transplantation could not be performed due to insufficient data on the exact timing of transplantation events in the cohort.Children diagnosed with HCM before the age of 1 year had a significantly higher frequency of death (*p* = 0.045, Fig. 2A). There was no significant difference for the other endpoints in children diagnosed before or after 1 year of age (Fig. 2B to E).

**Fig. 3:**
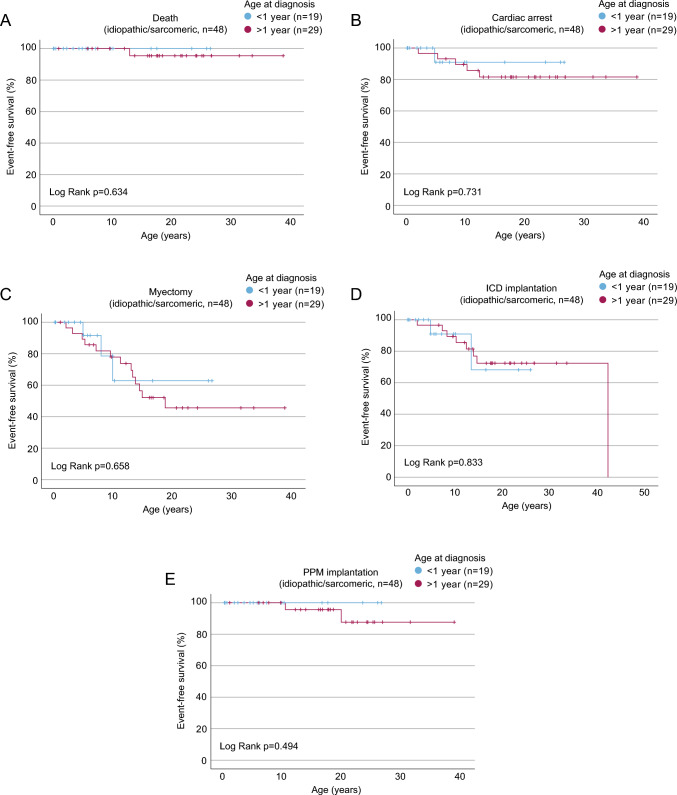
Outcome of pediatric idiopathic/sarcomeric HCM diagnosed before or after 1 year of age. Hazard free survival by Kaplan–Meier analysis for the endpoints **A** death, **B** cardiac arrest, **C** myectomy, **D** ICD implantation, or **E** PPM implantation for the subgroup “idiopathic/sarcomeric”, stratified by age at diagnosis < 1 or > 1 year of age. Exact information of date at diagnosis was available for 128 children, *n* = 19 < 1 year and *n* = 29 > 1 year. Patients without exact information on age at diagnosis were excluded from the analysis. Statistical analysis represents the p-value of a log-rank test

**Fig. 4:**
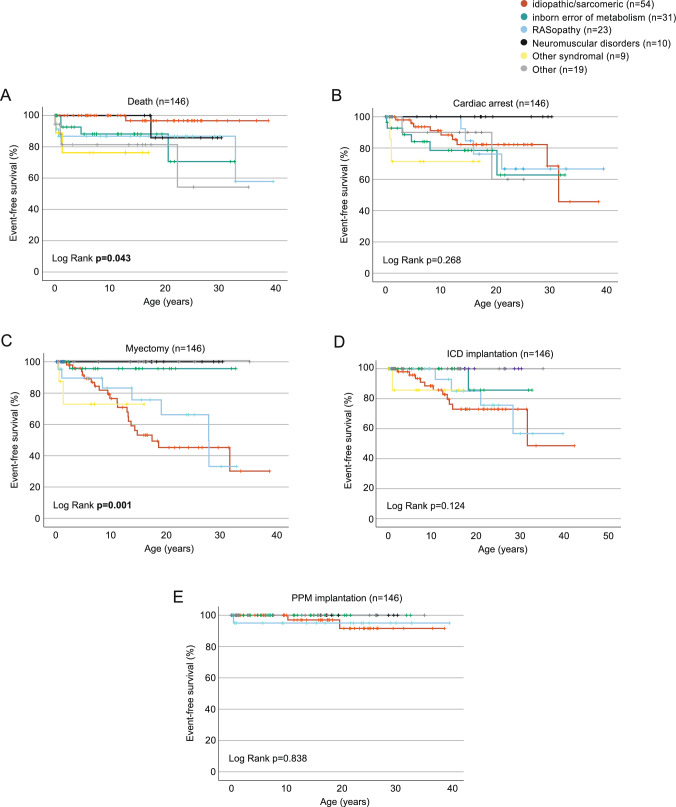
Outcome of pediatric HCM by underlying etiology. Hazard free survival by Kaplan–Meier analysis for the endpoints **A** death, **B** cardiac arrest, **C** myectomy, **D** ICD implantation, or **E** PPM implantation, stratified by the underlying etiology. Patient numbers are shown in each panel. Statistical analysis represents the p-value of a log-rank test. *p* values reaching the pre-defined statistical significance threshold of 0.05 are printed in bold

Since children diagnosed < 1 year more frequently included patients with non-idiopathic/non-sarcomeric forms of HCM, in which the analyzed endpoints may occurred more frequently, we conducted a subgroup analysis restricted to idiopathic/sarcomeric HCM diagnosed before or after 1 year of age (Fig. 3). There were no statistical differences by log-rank test for the analyzed endpoints death, cardiac arrest, myectomy, ICD or PPM implantation in idiopathic/sarcomeric HCM by age at diagnosis. These findings suggest that differences in outcomes by age at diagnosis are primarily driven by underlying etiology rather than age alone. We, therefore, performed Kaplan–Meier analysis to evaluate outcomes based on the underlying etiology (Fig. 4). The time by which the endpoints were reached differed significantly between subgroups for the endpoints myectomy and death (Log Rank test: *p* = 0.001 and *p* = 0.043, respectively) (Fig. 4A and B). Further pairwise comparisons of the subgroups revealed the underlying group differences, shown in Supplementary Table 4 and 5. No significant differences between subgroups were observed for the endpoints cardiac arrest (*p* = 0.268), ICD implantation (*p* = 0.124), and PPM implantation (*p* = 0.838) (Fig. 4C to E).

In addition, we assessed whether the usage of various drug classes was associated with altered outcome. The medication data were collected at the time of diagnosis and at the last clinical presentation, providing a longitudinal overview of prescribed drugs. For further analysis, medications were categorized into their respective drug classes. Class II and Class IV antiarrhythmics were the most frequently prescribed drugs, administered to 62 patients (42.5%) and 31 patients (21.2%), respectively. All other drug classes were prescribed less frequently and were not included in further analyses due to limited statistical power (Supplementary Table 6).

Among the prescribed medications, use of Class IV antiarrhythmics was associated with less frequent occurrence of the endpoint of death in Kaplan–Meier analysis (*p* = 0.030) (Supplementary Table 7). The subgroup analysis indicated that patients with idiopathic/sarcomeric HCM were most frequently prescribed Class IV antiarrhythmics, however, no significant differences were observed between and among individual subgroups. Similarly, Cox regression analysis stratified by subgroup did not reach statistical significance (*p* = 0.141), likely due to the limited number of cases (Supplementary Table 8). Nevertheless, in this cohort, Class IV antiarrhythmics were associated with a lower risk of death.

Although Class II antiarrhythmics did not reach statistical significance, likely due to an insufficient number of events for adequate power, a trend towards less frequent occurrence of the endpoint death was observed, as described previously [15].

### Causes of death

Given the significant differences in mortality based on the underlying etiology in pediatric HCM, we next examined causes of death to determine whether the higher mortality rates of non-sarcomeric/non-idiopathic subgroups were driven by cardiovascular or non-cardiovascular death. The causes of death varied and were heterogeneous (Table 4). Among the 16 deaths, 37.5% (*n* = 6) were due to cardiogenic shock on noncardiogenic shock related to sepsis, 25% (*n* = 4) resulted from other non-cardiovascular causes, 18.8% (*n* = 3) of patients died from other cardiovascular death, 12.5% (*n* = 2) of patients missed clear information on the mode of death and 6.3% (*n* = 1) died from anoxic brain injury in respiratory failure. Overall, mortality in pediatric HCM was driven by both cardiovascular and non-cardiovascular causes. In our cohort, death in idiopathic/sarcomeric and RASopathy-associated HCM were predominantly cardiovascular-related, whereas in other etiologies, death was often due to the underlying disease or by an exacerbation of the cardiovascular phenotype during septic infection.

**Table 4:** Causes of death in pediatric hypertrophic cardiomyopathy

Group	Age at death (years)	Mode of death	Cause of death	Infection
Idiopathic/sarcomeric	12.88	Other cardiovascular death	Intra/postoperative	None
Inborn error of metabolism	1.12	Cardiogenic shock on noncardiogenic shock	Septic infectious	Community-acquired
Inborn error of metabolism	1.22	Anoxic brain injury/respiratory failure	Septic infectious	Community-acquired
Inborn error of metabolism	4.78	Unknown	Unknown	None
Inborn error of metabolism	20.57	Cardiogenic shock on noncardiogenic shock	Septic infectious	Vommunity-acquired
RASopathy	0.16	Cardiogenic shock on noncardiogenic shock	Septic infectious	Peripartal infection
RASopathy	0.27	Other cardiovascular death	Intra/postoperative	None
RASopathy	0.7	Other cardiovascular death	Intra/postoperative	None
RASopathy	32.77	Cardiogenic shock on noncardiogenic shock	Septic infectious	Nosocomial infection
Neuromuscular disorders	17.44	Other noncardiovascular death	Metabolic aggravation in newly diagnosed type 1 diabetes	None
Other syndromal	0.17	Other noncardiovascular death	Kidney failure of unknown underlying disease	None
Other syndromal	1.36	Cardiogenic shock on noncardiogenic shock	Septic infectious	Community-acquired
Other	0.07	Other noncardiovascular death	Liver failure of unknown underlying disease	None
Other	1.02	Other noncardiovascular death	Septic infectious	Nosocomial infection
Other	1.17	Cardiogenic shock on noncardiogenic shock	Septic infectious	Community-acquired
Other	22.25	Missing	Missing	None

### Predictors of outcome

We analyzed whether demographics, positive genetic testing, or echocardiographic findings at first presentation are predictors of outcome in pediatric HCM (Table 5). The predictive information was analyzed for the endpoints myectomy, ICD implantation, cardiac arrest, and death. No analysis could be performed for PPM implantation and heart transplantation. Neither parameter reached statistical significance for the prediction of the endpoints ICD implantation or cardiac arrest in our cohort. Ejection fraction (EF) and fractional shortening (FS) at presentation were predictive of future myectomy (EF: HR 0.929, *p* = 0.008; FS: HR 1.096, *p* = 0.008). In addition, ejection fraction at presentation was predictive of the endpoint death (HR 0.950, *p* = 0.032).

**Table 5:** Predictors of outcome in pediatric hypertrophic cardiomyopathy

Group	Myectomy			ICD			Cardiac Arrest			Death		
Parameter	B coefficient (± SE)	Hazard ratio (95% CI)	*P*-Value	B coefficient (± SE)	Hazard ratio (95% CI)	*P*-Value	B coefficient (± SE)	Hazard ratio (95% CI)	*P*-Value	B coefficient (± SE)	Hazard ratio (95% CI)	*P*-Value
Gender	0.895 (0.549)	2.45 (0.83–7.18)	0.103	0.395 (0.814)	1.48 (0.30–7.32)	0.628	1.360 (0.709)	3.90 (0.97–15.65)	0.055	0.205 (0.708)	1.23 (0.31–4.91)	0.772
Age	0.662 (0.677)	1.94 (0.51–7.31)	0.329	1.339 (1.311)	3.82 (0.29–49.84)	0.307	− 0.642 (0.729)	0.53 (0.13–2.20)	0.378	− 0.437 (0.763)	0.65 (0.14–2.88)	0.567
Family history	0.652 (0.637)	1.92 (0.55–6.68)	0.306	0.001 (1.163)	1.00 (0.10–9.78)	0.999	0.609 (0.849)	1.84 (0.35–9.71)	0.474	0.104 (1.104)	1.11 (0.13–9.66)	0.925
Cosanguin	0.329 (1.120)	1.40 (0.16–12.49)	0.769	N/A	N/A	N/A	− 0.012 (1.178)	0.99 (0.10–9.94)	0.992	0.638 (1.019)	1.89 (0.26–13.96)	0.531
Genetic testing	-0.867 (0.638)	0.42 (0.12–1.47)	0.174	− 0.768 (1.004)	0.46 (0.07–3.32)	0.445	− 0.628 (0.704)	0.53 (0.13–2.12)	0.372	1.538 (0.978)	4.65 (0.68–31.66)	0.116
EF	-0.074 (0.028)	0.93 (0.88–0.98)	**0.008**	− 0.038 (0.057)	0.96 (0.86–1.08)	0.504	− 0.013 (0.044)	0.99 (0.91–1.07)	0.760	− 0.051 (0.024)	0.95 (0.91–1.00)	**0.032**
FS	0.091 (0.035)	1.10 (1.02–1.17)	**0.008**	0.067 (0.071)	1.07 (0.93–1.23)	0.348	0.023 (0.053)	1.02 (0.92–1.14)	0.655	0.032 (0.034)	1.03 (0.97–1.10)	0.341
IVSd	0.016 (0.037)	1.02 (0.94–1.09)	0.67	0.014 (0.043)	1.01 (0.93–1.10)	0.751	− 0.032 (0.062)	0.97 (0.86–1.09)	0.600	0.068 (0.065)	1.07 (0.94–1.22)	0.294
LVPWd	0.044 (0.069)	1.05 (0.91–1.20)	0.523	− 0.153 (0.159)	0.86 (0.63–1.17)	0.333	− 0.115 (0.142)	0.89 (0.67–1.18)	0.417	0.016 (0.089)	1.02 (0.85–1.21)	0.858
LVIDd	-0.019 (0.110)	0.98 (0.79–1.22)	0.866	0.013 (0.219)	1.01 (0.66–1.56)	0.953	0.117 (0.145)	1.12 (0.84–1.49)	0.422	− 0.058 (0.147)	0.94 (0.71–1.26)	0.695

Significant differences (*p* < 0.05) are highlighted in bold

Due to the limited availability of diastolic echocardiographic parameters, their predictive value could only be analyzed in relation to a combined endpoint of death, ICD, PPM and myectomy. The analysis of diastolic dysfunction plotted against the time from HCM diagnosis to the first event, revealed no statistically significant differences for diastolic echo parameters in parameter distribution in this cohort, including in secondary analyses across subgroups (Supplementary Table 9).

In addition, we analyzed the predictive value of clinical laboratory parameters in pediatric HCM. All available laboratory test results from the patient records were incorporated for a predefined set of parameters, including: creatine kinase (CK), creatine kinase muscle–brain type (CK-MB), C-reactive protein (CRP), hemoglobin (Hb), hematocrit (Hct), high-sensitivity troponin T (hs-TnT), N-terminal prohormone of brain natriuretic peptide (NT-proBNP), lactate dehydrogenase (LDH), leukocyte count (Leuc), lactate (Lact), ferritin (Ferr), and transferrin saturation (TfSt). For each parameter, the maximum value recorded in the available clinical history was used for subsequent predictive analyses. Although not all parameters were measured with equal frequency across patients (Supplementary Table 10), elevated NT-proBNP emerged as the strongest individual predictor of death (*p* = 0.026, OR = 8.25), while elevated hs-TnT was significantly associated with patients undergoing myectomy (*p* = 0.004, OR = 4.50) (Supplementary Table 11). Neither of both parameters showed significant differences between subgroups. Other biomarkers, including CRP, leukocyte count, LDH, and CK-MB, demonstrated no statistically significant predictive value in this cohort.

## Discussion

We describe the clinical characteristics and outcomes of a large cohort of 146 patients diagnosed with HCM during childhood, stratified by underlying etiology. This study was conducted at a tertiary care center, which may limit generalizability to other healthcare settings. Our analysis revealed two distinct peaks in the age at diagnosis in children: infancy and around puberty, consistent with previous reports [3, 8]. A substantial proportion (53.9%) of patients were diagnosed before one year of age. Earlier diagnosis was associated with higher mortality, with 62.5% of deaths occurring within the first 2 years of life. Infant-onset HCM was attributed to a diverse range of etiologies, including idiopathic/sarcomeric, inborn errors of metabolism, RASopathies, and other syndromic or non-syndromic conditions. In contrast, HCM diagnosed after the age of three years was predominantly classified as idiopathic/sarcomeric or neuromuscular.

The clinical outcomes differed markedly based on the underlying etiology. In the neuromuscular subgroup, comprising exclusively Friedreich’s ataxia-associated pediatric HCM in our cohort, no cases of cardiac arrest were observed, and no patient obtained ICD placement. This findings are consistent with previous reports on the outcome of Friedreich´s ataxia-associated HCM in children [12, 16] revealing low risk of malignant ventricular arrhythmias or obstruction in these patients. Children with inborn error of metabolism exhibited more pronounced free wall hypertrophy on echocardiography, a pattern commonly associated with metabolic or infiltrative disease [17]. Pediatric idiopathic/sarcomeric HCM, RASopathy and other syndromal-associated HCM frequently underwent myectomy due to outflow tract obstruction. The children diagnosed with idiopathic/sarcomeric or neuromuscular HCM had a good overall outcome. The survival rates were markedly lower in patients with non-idiopathic/non-sarcomeric HCM compared to those with idiopathic/sarcomeric HCM. Consistent with previous studies [7, 8, 18], children diagnosed with HCM < 1 year of age had poorer outcomes compared to children diagnosed > 1 year of age. Since a sub-analysis of idiopathic/sarcomeric HCM cases found no difference in outcome based on age at diagnosis, the poorer outcome of HCM in infants is likely attributable to the higher incidence of non-idiopathic/non-sarcomeric HCM in this age group. Importantly, patients diagnosed < 1 year of age who survived beyond the first 2 years of life demonstrated an overall good survival, showing similar long-term outcomes compared to patients diagnosed > 1 years of age.

Among the cohort, 11% (*n* = 16) of patients died during follow-up, with the majority of deaths occurring within the first two years of life. The causes of death were heterogeneous but frequently involved cardiorespiratory failure in response to an infection or were caused by an underlying disease. In contrast, death due to primary cardiac failure was less common. These findings underscore the importance of comprehensive management of non-cardiac symptoms and complications in pediatric HCM, particularly in younger children, where syndromic forms are more prevalent.

Neither demographic factors nor positive genetic testing predicted outcome in this cohort. Among the baseline echocardiographic parameters, only ejection fraction and fractional shortening showed some significant predictive value. Other echocardiographic parameters such as diastolic function showed some non-significant trend for predictive value, however, the limited availability of these data precluded definitive conclusions. The prescription of Class IV antiarrhythmics was associated with improved survival during follow-up, underscoring the importance of individualized pharmacological management. Elevated NT-proBNP emerged as the strongest independent laboratory parameter that predicted mortality. These results support the integration of biomarker evaluation and tailored pharmacotherapy in managing children with HCM. Thus, the main determinant of the outcome appears to be underlying etiology, infant-onset and cardiac contractility by echocardiography, and NT-proBNP, as previously described in a risk stratification analysis of the pediatric cardiomyopathy registry [6].

Our analysis highlights significant differences in the clinical course and prognosis among subgroups of pediatric HCM. With recent advancements in prevention and targeted therapies for HCM and the emergence of novel treatments aimed at addressing the underlying mechanisms of HCM-causing etiologies [19–25], our findings underscore the critical need for personalized management strategies tailored to childhood-onset HCM. 

## Methods

### Patient identification and data collection

The patients clinically diagnosed with hypertrophic cardiomyopathy (HCM) before the age of 18 at Heidelberg University Hospital, Germany (2000–2024) were included (*n* = 146). The clinical data were obtained from the Medical Data Integration Center (Institute of Medical Informatics, Heidelberg University) and supplemented by manual extraction from patient records. The dataset comprised demographic characteristics, clinical history genetic findings, clinical laboratory results and echocardiographic parameters. Patients were identified through two sources:

1. The Medical Data Integration Center (Institute of Medical Informatics, Heidelberg University), where cases were classified based on automated data extraction, including ICD codes and diagnostic keywords. Automatically extracted data was manually evaluated and corrected based on clinical data where necessary.
2. Manual extraction for patients attending the pediatric cardiology outpatient clinic who had not been extracted by the Medical Data Integration Center.
3. To ensure high quality and consistency of the data, the extracted cases were subjected to an interrater reliability check in which two independent reviewers (F.M. and C.S.) reassessed the clinical data and disease classification.

### Subgroup classification

The patients were categorized into six major subgroups based on underlying etiology, genetic findings, and clinical phenotype:

- *Idiopathic/sarcomeric*: patients with pathogenic or likely pathogenic variants in sarcomeric genes with definitive or strong evidence of gene-disease validity for hypertrophic cardiomyopathy in ClinGen (02/2025). Patients were classified as **“**idiopathic**”** if no signs of other underlying HCM-causing disease were present in the patient’s clinical history or genetic testing.
- *Inborn error of metabolism-associated HCM*: patients with HCM secondary to metabolic disorders (including mitochondrial diseases) confirmed by biochemical, enzymatic, or genetic testing, as well as clinical diagnosis from an expert tertiary center.
- *RASopathy-associated HCM*: patients with HCM secondary to genetically or clinically diagnosed Noonan syndrome, Noonan syndrome with multiple lentigines, Costello syndrome, cardiofaciocutaneous syndrome, and Noonan syndrome with loose anagen hair.
- *Neuromuscular disease-associated HCM*: patients with HCM secondary to neuromuscular disorders, including Friedreich ataxia in our cohort.
- *Other syndromic-associated HCM*: patients with HCM and a syndromic disease other than inborn errors of metabolism, RASopathy or neuromuscular disease.
- *Other*: patients who met diagnostic criteria for HCM and did not fit into the above categories.

Subgroup assignment was conducted manually based on clinical history, genetic findings, and diagnostic criteria derived from the MEDIC analysis.

### Statistical analysis

Data visualization was performed using IBM SPSS Statistics (Version 29.0.2.0, Chicago, Illinois, USA) and GraphPad Prism 7.0. Categorical variables were expressed as percentages and compared using the Pearson Chi-Square test or Fisher’s exact test. Continuous variables were summarized as means with 95% confidence intervals for normally distributed data or as medians with minimum–maximum ranges for non-normally distributed data. Comparisons between subgroups were conducted using an independent t-test for normally distributed variables. For time-to-event analyses, the Kaplan–Meier method was used to estimate survival and major clinical outcomes, including myectomy, ICD implantation, PPM implantation, heart transplantation, and cardiac arrest. Comparisons between subgroups were performed using the log-rank test. Cox proportional hazards regression was applied to identify predictors of outcomes, adjusting for relevant covariates. Logistical regression was applied to clinical data to find independent predictors for endpoints. Kruskal–Wallis-Test was used to analyze differences between subgroups for continuous, ordinally distributed parameters. Post-hoc analysis was performed using the Mann–Whitney-U-Test. The Friedman test was used to analyze differences in blood test parameters across multiple time points. Data was censored at the time of the event or the latest available follow-up. The missing data were handled by case-wise exclusion, and no specific imputation techniques were applied.

## Supplementary Information

Below is the link to the electronic supplementary material.

Supplementary Table 1: Patient classification. Supplementary file3 (XLSX 13 KB)

Supplementary Table 2: Genetic testing frequency. Supplementary file2 (XLSX 14 KB)

Supplementary Table 3: Genetic testing results. Supplementary file3 (XLSX 38 KB)

Supplementary Table 4: Myectomy Pairwise Comparisons. Supplementary file4 (XLSX 11 KB)

Supplementary Table 5: Death Pairwise Comparisons. Supplementary file5 (XLSX 13 KB)

Supplementary Table 6: Drug Frequency. Supplementary file6 (XLSM 11 KB)

Supplementary Table 7: Outcome Class IV Antiarrhythmics. Supplementary file7 (XLSX 12 KB)

Supplementary Table 8: Outcome Class IV Antiarrhythmics by Subgroup. Supplementary file8 (XLSM 11 KB)

Supplementary Table 9: Additional Echocardiography results. Supplementary file9 (XLSM 10 KB)

Supplementary Table 10: Clinical Laboratory Parameters. Supplementary file10 (XLSM 23 KB)

Supplementary Table 11: Predictors Clinical Laboratory. Supplementary file11 (XLSX 10 KB)

## Data Availability

Anonymized data supporting the findings of this study are available from the corresponding author upon reasonable request.
